# A Comparative Assessment of Hand Preference in Captive Red Howler Monkeys, *Alouatta seniculus* and Yellow-Breasted Capuchin Monkeys, *Sapajus xanthosternos*

**DOI:** 10.1371/journal.pone.0107838

**Published:** 2014-10-01

**Authors:** Nasibah Sfar, Madhur Mangalam, Werner Kaumanns, Mewa Singh

**Affiliations:** 1 Department of Biology, University of Cologne, Cologne, Germany; 2 Biopsychology Laboratory and Institution of Excellence, University of Mysore, Mysore, India; 3 LTM Research and Conservation, Gleichen, Germany; 4 Evolutionary & Organismal Biology Unit, Jawaharlal Nehru Centre for Advanced Scientific Research, Bangalore, India; CNR, Italy

## Abstract

There are two major theories that attempt to explain hand preference in non-human primates–the ‘task complexity’ theory and the ‘postural origins’ theory. In the present study, we proposed a third hypothesis to explain the evolutionary origin of hand preference in non-human primates, stating that it could have evolved owing to structural and functional adaptations to feeding, which we refer to as the ‘niche structure’ hypothesis. We attempted to explore this hypothesis by comparing hand preference across species that differ in the feeding ecology and niche structure: red howler monkeys, *Alouatta seniculus* and yellow-breasted capuchin monkeys, *Sapajus xanthosternos*. The red howler monkeys used the mouth to obtain food more frequently than the yellow-breasted capuchin monkeys. The red howler monkeys almost never reached for food presented on the opposite side of a wire mesh or inside a portable container, whereas the yellow-breasted capuchin monkeys reached for food presented in all four spatial arrangements (scattered, on the opposite side of a wire mesh, inside a suspended container, and inside a portable container). In contrast to the red howler monkeys that almost never acquired bipedal and clinging posture, the yellow-breasted capuchin monkeys acquired all five body postures (sitting, bipedal, tripedal, clinging, and hanging). Although there was no difference between the proportion of the red howler monkeys and the yellow-breasted capuchin monkeys that preferentially used one hand, the yellow-breasted capuchin monkeys exhibited an overall weaker hand preference than the red howler monkeys. Differences in hand preference diminished with the increasing complexity of the reaching-for-food tasks, i.e., the relatively more complex tasks were perceived as equally complex by both the red howler monkeys and the yellow-breasted capuchin monkeys. These findings suggest that species-specific differences in feeding ecology and niche structure can influence the perception of the complexity of the task and, consequently, hand preference.

## Introduction

Typically, non-human primates preferentially use one hand to perform a unimanual task, or to execute the most complex action while performing a bimanual task [1]. Several studies and meta-analyses suggest that hand preference in non-human primates is likely to be a precursor of population-level right-hand preference in humans [2]–[4] (although there are a few studies and meta-analyses that advocate the opposite [5], [6]). Thus, understanding the forms and functions of hand preference in non-human primates may help in reconstructing the evolutionary origin of population-level right-hand preference in humans and in explaining the adaptive values of the underlying cerebral lateralization. Conflicting findings on hand preference in non-human primates, however, have complicated the process of reconstructing the evolutionary origin of hand preference. The source of these complications are several individual-specific traits, such as age-class, sex, experience, and external factors, such as body posture and task complexity, which have been reported to heterogeneously influence hand preference [5]–[7].

There are two major theories that attempt to explain hand preference in non-human primates–the ‘task complexity’ theory [1] and the ‘postural origins’ theory [2]. The task complexity theory proposes that the strength of hand preference increases with increasing task complexity as novelty and practice variables influence manual asymmetries. Consistent with the task complexity theory, studies on several non-human primate species reported that the same individuals that used both hands to a similar extent in simple unimanual reaching-for-food tasks (here, ‘reaching-for-food’ refers to the manual action involved in obtaining food using hand(s)/mouth), preferentially used one hand in the relatively complex bimanual tasks (red-capped mangabeys, *Cercocebus torquatus* [8], vervet monkeys, *Chlorocebus aethiops* [9], brown capuchin monkeys (now known as *Sapajus apella* [10]) [11], white-faced capuchin monkeys, *Cebus capucinus* [12], olive baboons, *Papio anubis* [13], Campbell's monkeys, *Cercopithecus campbelli* [14], De Brazza's monkeys, *Cercopithecus neglectus* [15], Sichuan snub-nosed monkeys, *Rhinopithecus roxellana* [16], rhesus macaques, *Macaca mulatta* [17], gorillas, *Gorilla gorilla berenge*i [18], and chimpanzees, *Pan troglodytes* [19]). Alternatively, the postural origins theory proposes that hand preference in non-human primates evolved owing to structural and functional adaptations to foraging in arboreal contexts. Initially, for some unknown reason the left hand became specialized for visually guided movements, and the right hand became specialized for postural support. Accordingly, studies on several non-human primate species reported that the same individuals that used both hands to a similar extent in unimanual reaching-for-food tasks that require tripedal or quadrupedal posture, preferentially used the left hand in those tasks that require bipedal posture (lesser bushbabies, *Galago senegalensis* [20], ruffed lemurs, *Varecia variegata* [21], and sifakas, *Propithecus* spp. [22]). Thereon, with the evolution of a lesser arboreal lifestyle, the right hand was no longer required to obtain postural support and became specialized for manipulating objects while coordinating complex bimanual actions. In this respect, studies on several non-human primate species reported that the same individuals that used both hands to a similar extent in unimanual reaching-for-food tasks that require tripedal/quadrupedal posture, preferentially used the right hand in those tasks that require bipedal posture (red-capped mangabeys [8], tufted capuchin monkeys [23], bonobos, *Pan paniscus* [24], chimpanzees, and orangutans, *Pongo pygmaeus* [25]). In a nutshell, the task complexity theory incorporates the physical constraints imposed by tasks, whereas the postural origins theory incorporates the physical constraints imposed by body postures. These different types of physical constraints, however, may not necessarily elicit mutually consistent hand preferences. Besides, many other factors that these two theories do not consider could possibly have played a role in the evolution of hand preference in non-human primates, as well as in humans [5], [26].

In the present study, we proposed a third hypothesis to explain the evolutionary origin of hand preference in non-human primates. In line with the ideas put forward by Parker and Gibson [27] and Westergaard [28], we hypothesized that hand preference in non-human primates could have evolved owing to structural and functional adaptations to feeding, from here on referred to as the ‘niche structure’ hypothesis. We attempted to explore this hypothesis by comparing hand preference between species that differ in the feeding ecology and niche structure, namely red howler monkeys, *Alouatta seniculus* and yellow-breasted capuchin monkeys (formerly, *Cebus xanthosternos*, but now known as *Sapajus xanthosternos* [10]). Red howler monkeys and yellow-breasted capuchin monkeys occupy different feeding niches owing to their more arboreal or terrestrial lifestyles. Wild red howler monkeys mostly feed on food resources that are very simple to process (e.g., barks, leaves, unripe fruits, termitarium soil, and moss) [29], [30]; they frequently hang upside down using the tail and then use the mouth for feeding purposes [31]. On the other hand, yellow-breasted capuchin monkeys frequently use one or both hands in a coordinated manner while performing diverse, complex manipulative activities [32], including tool use [33]. Comparing hand- or mouth-usage patterns between these two species, we expected that the red howler monkeys, but not the yellow-breasted capuchin monkeys, would use the mouth more frequently than the hands for feeding purposes. As yellow-breasted capuchin monkeys habitually manipulate physical objects [31], we expected that they would perceive a particular reaching-for-food task as less complex than the red howler monkeys and, consequently, would also exhibit an overall weaker hand preference than the red howler monkeys. However, we expected that these differences would diminish with the increasing complexity of the reaching-for-food tasks (imposed by the increasing mobility of the food-containing substrates) as relatively more complex tasks are likely to be perceived as equally complex by both the red howler monkeys and the yellow-breasted capuchin monkeys.

## Methods

### Ethics Statement

Our research was completely non-invasive. All experimental work adhered to the American Society of Primatologists “Principles for the Ethical Treatment of Non-Human Primates,” approved in 2001. Whereas we did not approach any Institutional Animal Care and Use Committee, the present study was approved by the Director of the Cologne Zoo.

### Subjects and Housing Conditions

The subjects were (a) 12 red howler monkeys: 3 adult males, 1 infant male, 5 adult females, 2 juvenile females, and 1 infant female; and (b) 7 yellow-breasted capuchin monkeys: 1 adult male, 1 juvenile male, 1 infant male, 2 adult females, 1 juvenile female, and 1 infant female. Subjects were housed in indoor-outdoor enclosures that were enriched with objects such as branches, ladders, ropes, cloth bags, and toys, at the Cologne Zoo, Germany (see Sfar [34] for further details on housing conditions). Both the red howler monkeys and the yellow-breasted capuchin monkeys were provided with food material in variable spatial arrangements at their regular feeding times; water was available *ad libitum*.

### Reaching-for-Food Tasks

**Figure 1:**
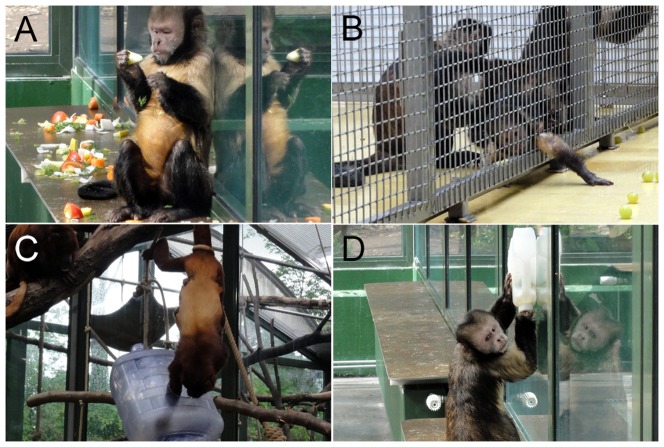
A red howler monkey and a yellow-breasted capuchin monkeys retrieving food in the different reaching-for-food tasks. A yellow-breasted capuchin monkey reaching for food scattered on a surface (A) and presented on the opposite side of a wire mesh (B). A red howler monkey reaching for food presented inside a suspended container (C). The yellow-breasted capuchin monkey reaching for food presented inside a portable container (D).

We observed how the red howler monkeys and the yellow-breasted capuchin monkeys used hand and mouth across different types of reaching-for-food activities. These activities required: (a) reaching for small-sized fruits and vegetables scattered on the ground (Figure 1A), or reaching for food presented (b) on the opposite side of a wire mesh through a small slit (Figure 1B), (c) inside a suspended container (i.e., a gallon water bottle) (Figure 1C), or (d) inside a portable container (e.g., a gallon water bottle or a corrugated paperboard carton) (Figure 1D), following an increasing order of complexity in terms of the maneuvering dexterity required to obtain the food, because of the increasing mobility of the food-containing substrates. We presented each task to the entire group in separate sessions and for each task we carried out a variable number of sessions for the red howler monkeys (scattered: n = 89; opposite side of a wire mesh: n = 3; inside a suspended container: n = 16; inside a portable container: n = 3; we did not conduct more trials of the second and the fourth tasks for the red howler monkeys as they almost never reached for food presented on the opposite side of a wire mesh or inside a portable container) and the yellow-breasted capuchin monkeys (scattered: n = 11; opposite side of a wire mesh: n = 16; inside a suspended container: n = 36; inside a portable container: n = 13).

### Data Collection

We used the continuous-focal-animal-sampling method [35] with 10 min focal sessions. During a focal session we video recorded the feeding behavior of the focal red howler monkey or the yellow-breasted capuchin monkey. Additionally, NS scored the recorded videos to obtain the hand and mouth usage by the monkeys across the different types of reaching-for-food activities, and categorized the body posture acquired by the monkeys while retrieving food as: (a) sitting – body supported by the buttocks and the back upright (with the two hindlimbs potentially providing additional postural support), (b) bipedal – body supported by the two hindlimbs, (c) tripedal – body supported by three limbs (typically, a forelimb and the two hindlimbs), (d) clinging – body adhered to a vertical surface by three limbs (typically, a forelimb and the two hindlimbs), or (e) hanging – body suspended upside down by the tail or limb(s). In order to preserve the independence of each single manual action, i.e., a bout, NS started a new observation after the subject changed body posture or shifted to some other activity, and discarded the observations in which the manual actions could have been conditioned by the presence of conspecific(s).

### Statistical Analyses

We determined an overall z-score for each of the red howler monkeys and the yellow-breasted capuchin monkeys using the formula: z-score  =  [R – (R+L)/2]/√[(R+L)/4] (where ‘R’ and ‘L’ represent the frequency of usage of the right and left hand respectively). We used the obtained z-scores to determine hand preferences (z≤−1.96: left-hand preference; −1.96<z<1.96: no hand preference; z≥1.96: right-hand preference).

We determined the handedness index (HI) values (overall, per reaching-for-food activity, and per body posture) for each of the red howler monkeys and the yellow-breasted capuchin monkeys, using the formula: HI =  (R – L)/(R+L). The obtained HI values ranged from −1 to +1, with positive values indicating a bias towards right-hand use and negative values indicating a bias towards left-hand use, while the absolute HI values indicate the strength of the bias.

We used two-tailed tests and performed all statistical analyses on SPSS 20. Since our data were normally distributed and variances were homogeneous, we used parametric tests. We considered the outcomes of the tests significant whenever the value of alpha was lower than 0.05.

## Results

**Figure 2:**
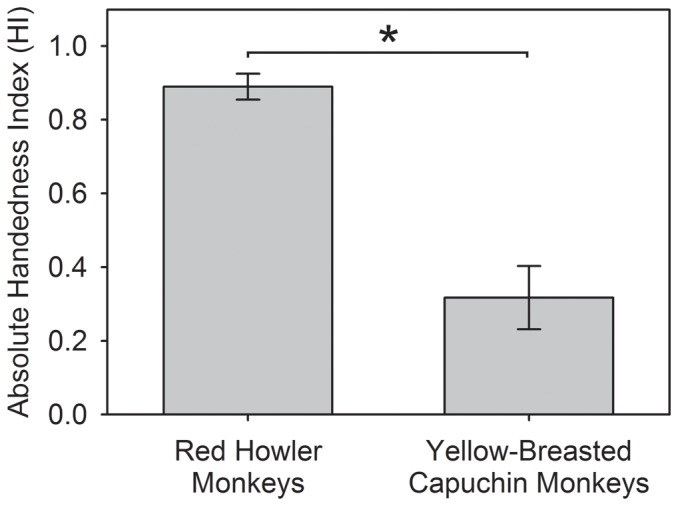
The overall mean ± se HI values for the red howler monkeys (n  =  12) and the yellow-breasted capuchin monkeys (n  =  7). *p <0.050.

Table 1 describes the overall hand and mouth usage for the red howler monkeys and the yellow-breasted capuchin monkeys. We carried out a mean (±sd) of 258 (±175) observations per subject for the red howler monkeys (n = 12; range: 37 to 596) and 369 (±151) for the yellow-breasted capuchin monkeys (n = 7; range: 205 to 588). The red howler monkeys used the mouth more frequently than the yellow-breasted capuchin monkeys (red howler monkeys: mean ±se  = 42.750±7.810, yellow-breasted capuchin monkeys: 7.429±6.267; independent samples t-test: t = 3.109, df = 17, p = 0.006). There was no difference between the proportion of the red howler monkeys and the yellow-breasted capuchin monkeys that preferentially used one hand (red howler monkeys: 12/12, yellow-breasted capuchin monkeys: 6/7; Fisher's exact test: p = 0.368), but the overall absolute HI values were higher for the red howler monkeys than for the yellow-breasted capuchin monkeys (independent samples t-test: t = 7.205, df = 17, p<0.001) (Figure 2). Individual differences in the number of observations did not skew the distribution of HI values as there was no correlation between the number of observations of hand usage and the absolute HI value, both for the red howler monkeys (Spearman's rank correlation: r_s_ = 0.116, n = 12, p = 0.720) and the yellow-breasted capuchin monkeys (Spearman's rank correlation: r_s_ = 0.357, n = 7, p = 0.432).

**Table 1:** The Overall Hand and Mouth Usage for the Red Howler Monkeys and the Yellow-Breasted Capuchin Monkeys.

Species	Age-Class	Sex	Hand Usage		Mouth Usage	HI	z-score	Hand Preference^a^
Individual			L	R				
Red Howler Monkeys								
Pakoa	Adult	Male	2	128	13	0.969	11.051	Right
Mikey	Adult	Male	229	1	91	−0.991	−15.034	Left
Boxer	Adult	Male	0	114	25	1.000	10.678	Right
Arun	Infant	Male	49	3	46	−0.885	−6.379	Left
Negrita	Adult	Female	0	69	8	1.000	8.307	Right
Tchona	Adult	Female	27	176	44	0.734	10.458	Right
Runa	Adult	Female	15	319	76	0.910	16.634	Right
Ymanoe	Adult	Female	3	548	45	0.989	23.218	Right
Kamoi	Adult	Female	28	207	30	0.762	11.677	Right
Namid	Juvenile	Female	212	14	33	−0.876	−13.171	Left
Talutah	Juvenile	Female	14	420	81	0.935	19.489	Right
Guma	Infant	Female	3	13	21	0.625	2.500	Right
Yellow-Breasted Capuchin Monkeys								
Ayukah	Adult	Male	69	425	1	0.721	16.017	Right
Cigala	Juvenile	Male	265	192	2	−0.160	−3.415	Left
Niken	Infant	Male	88	116	1	0.137	1.960	Right
Ibama	Adult	Female	166	55	1	−0.502	−7.467	Left
Riley	Adult	Female	401	185	2	−0.369	−8.923	Left
Tenya	Juvenile	Female	184	149	45	−0.105	−1.918	None
Nashota	Infant	Female	146	92	0	−0.227	−3.500	Left

‘R’ and ‘L’ indicate the frequency of usage of the left and right hand respectively;

a z≤−1.96: left-hand preference; −1.96<z<1.96 no hand preference; z≥1.96: right-hand preference.

**Figure 3:**
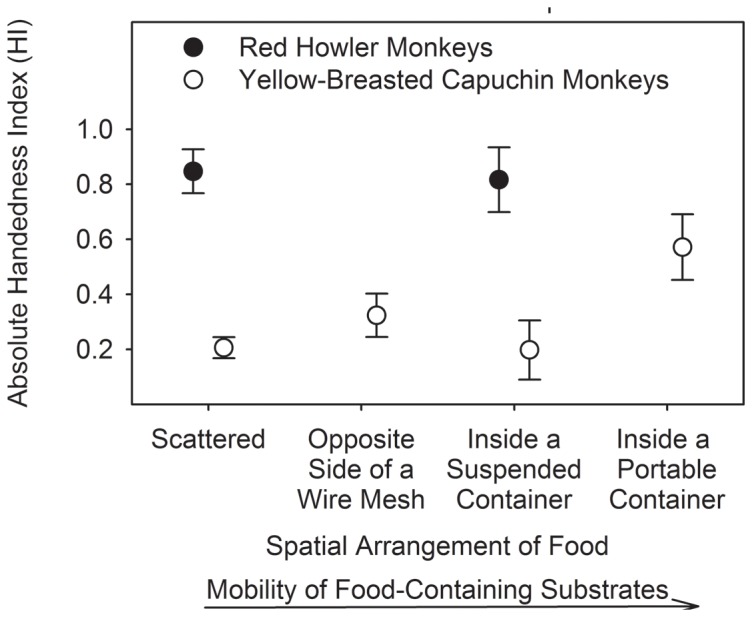
Mean ± se HI values per reaching-for-food task for the red howler monkeys (scattered: n  =  12; inside a suspended container: n  =  9) and the yellow-breasted capuchin monkeys (scattered: n  =  6; on the opposite side of a wire mesh, inside a suspended container, inside a portable container: n  =  7). Vertical bars indicate SE.

Table 2 describes the hand usage across the different reaching-for-food tasks for the red howler monkeys and the yellow-breasted capuchin monkeys. The red howler monkeys had higher absolute HI values than the yellow-breasted capuchin monkeys whenever the food was scattered (independent samples t-test: t = 5.864, df = 16, p<0.001) or presented inside a suspended container (independent samples t-test: *t* = 3.554, df = 12, p = 0.004) (Figure 3). The other comparisons were not feasible because of insufficient data on the red howler monkeys, which almost never reached for food presented either on the opposite side of a wire mesh or inside a portable container. Whereas there was no difference in the absolute HI values for the red howler monkeys between the two reaching-for-food tasks (independent samples t-test: t = 0.601, df = 17, p = 0.556), there was a difference in the absolute HI values for the yellow-breasted capuchin monkeys between the four reaching-for-food tasks (one-way ANOVA: F = 3.905, df = 3, 23, p = 0.022). Fisher's LSD post-hoc test revealed that the absolute HI values for the yellow-breasted capuchin monkeys were higher when the food was presented inside a portable container than when scattered (p = 0.000) or presented inside a suspended container (p = 0.009) (Figure 3).

**Table 2:** Hand Usage across the Different Reaching-for-Food Activities for the Red Howler Monkeys and the Yellow-Breasted Capuchin Monkeys.

Species	Scattered			Opposite Side of a Wire Mesh			Inside a Suspended Container				Inside a Portable Container		
Individual	L	R	HI	L	R	HI	L	R	HI		L	R	HI
Red Howler Monkeys													
Pakoa	2	93	0.958	-	-	-	0	35	1.000		-	-	-
Mikey	222	1	−0.991	-	-	-	3	0	−1.000		4	0	−1.000
Boxer	0	114	1.000	-	-	-	0	0	-		-	-	-
Arun	49	3	−0.885	-	-	-	0	0	-		-	-	-
Negrita	0	68	1.000	-	-	-	0	1	1.000		-	-	-
Tchona	20	171	0.791	-	-	-	7	5	−0.167		-	-	-
Runa	10	203	0.906	-	-	-	5	116	0.917		-	-	-
Ymanoe	3	473	0.987	0	7	1.000	0	66	1.000		0	2	1.000
Kamoi	16	158	0.816	-	-	-	12	49	0.607		-	-	-
Namid	181	14	−0.856	-	-	-	31	0	−1.000		-	-	-
Talutah	4	363	0.978	0	5	1.000	10	49	0.661		0	3	1.000
Guma	13	13	0.000	-	-	-	0	0	-		-	-	-
Yellow-Breasted Capuchin Monkeys													
Ayukah	47	66	0.168	11	80	0.758	9	89	0.816		2	190	0.979
Cigala	17	18	0.029	96	65	−0.193	94	92	−0.011		58	17	−0.547
Niken	7	5	−0.167	21	40	0.311	55	58	0.027		5	13	0.444
Ibama	26	19	−0.156	4	8	0.333	15	12	−0.111		121	16	−0.766
Riley	44	23	−0.313	110	89	−0.106	99	63	−0.222		148	10	−0.873
Tenya	8	10	0.111	87	53	−0.243	65	68	0.023		24	18	−0.143
Nashota	1	3	0.500	82	42	−0.323	60	42	−0.176		3	5	0.250

‘R’ and ‘L’ indicate the frequency of usage of the left and right hand respectively.

**Figure 4:**
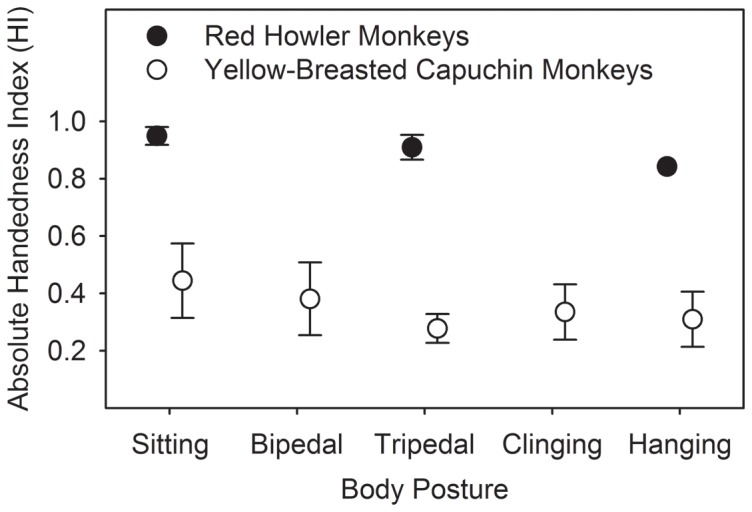
Mean ± se HI values per body posture for the red howler monkeys (sitting: n  =  9; tripedal, hanging: n  =  11) and the yellow-breasted capuchin monkeys (sitting and tripedal: n  =  7; bipedal, clinging, hanging: n  =  6). Vertical bars indicate SE.

Table 3 describes the hand usage according to the different body postures acquired by the red howler monkeys and the yellow-breasted capuchin monkeys while reaching for food. For the analysis, we considered only those postures for which we had at least 5 observations on at least 6 subjects (red howler monkeys: sitting, tripedal, and hanging; yellow-breasted capuchin monkeys: sitting, bipedal, tripedal, clinging, and hanging). There was no difference in the absolute HI values with respect to the different body postures for both the red howler monkeys (one-way ANOVA: F = 0.915, df = 2, 28, p = 0.412) and the yellow-breasted capuchin monkeys (one-way ANOVA: F = 0.494, df = 4, 27, p = 0.740) (Figure 4).

**Table 3:** Hand Usage According to the Different Body Postures Acquired by the Red Howler monkeys and the Yellow-Breasted Capuchin monkeys.

Species	Sitting			Bipedal			Tripedal			Clinging			Hanging		
Individual	L	R	HI	L	R	HI	L	R	HI	L	R	HI	L	R	HI
Red Howler Monkeys															
Pakoa	0	0	-	0	0	-	0	14	1.000	0	0	-	2	114	0.966
Mikey	213	1	−0.991	0	0	-	8	0	−1.000	0	0	-	8	0	−1.000
Boxer	0	103	1.000	0	0	-	0	11	1.000	0	0	-	0	0	-
Arun	0	0	-	0	0	-	40	3	−0.860	0	0	-	9	0	−1.000
Negrita	0	31	1.000	0	0	-	0	33	1.000	0	0	-	0	5	1.000
Tchona	18	129	0.755	0	0	-	1	37	0.947	0	0	-	8	10	0.111
Runa	0	19	1.000	0	4	1.000	3	114	0.949	0	0	-	12	182	0.876
Ymanoe	1	269	0.993	0	3	1.000	1	189	0.989	0	0	-	1	87	0.977
Kamoi	1	0	−1.000	0	0	-	6	21	0.556	0	0	-	21	186	0.797
Namid	20	2	−0.818	0	0	-	96	11	−0.794	0	0	-	96	1	−0.979
Talutah	1	176	0.989	0	0	-	0	57	1.000	0	1	1.000	13	186	0.869
Guma	0	0	-	0	0	-	1	2	0.333	0	0	-	2	11	0.692
Yellow-Breasted Capuchin Monkeys															
Ayukah	37	307	0.785	2	51	0.925	26	59	0.388	2	4	0.333	2	4	0.333
Cigala	137	112	−0.100	35	26	−0.148	19	18	−0.027	61	26	−0.402	13	10	−0.130
Niken	46	78	0.258	1	4	0.600	8	4	−0.333	15	20	0.143	18	10	−0.286
Ibama	120	38	−0.519	2	5	−0.600	26	12	−0.368	0	0	-	0	0	-
Riley	237	98	−0.415	32	26	−0.103	42	21	−0.333	75	38	−0.327	15	2	−0.765
Tenya	90	70	−0.125	20	11	−0.290	13	18	0.161	40	21	−0.311	21	29	0.160
Nashota	67	50	−0.145	2	2	0.000	5	10	0.333	56	19	−0.493	16	11	−0.185

‘R’ and ‘L’ indicate the frequency of usage of the left and right hand respectively.

## Discussion

Species that differ in the feeding ecology and niche structure are likely to have diverse behavioral repertoires allowing them to respond appropriately to different ecological challenges (or the same challenge to variable degrees) (see, for example, interspecific comparisons on self-control tasks [36], [37]). In similar contexts, species may, therefore, behave differently. In the present study (a) the red howler monkeys used the mouth to obtain food more frequently than the yellow-breasted capuchin monkeys, (b) the red howler monkeys almost never reached for food presented on the opposite side of a wire mesh or inside a portable container, whereas the yellow-breasted capuchin monkeys reached for food presented in all four spatial arrangements (scattered, on the opposite side of a wire mesh, inside a suspended container, and inside a portable container), and (c) in contrast to the red howler monkeys that almost never acquired bipedal and clinging posture, the yellow-breasted capuchin monkeys acquired all five body postures (sitting, bipedal, tripedal, clinging, and hanging). Although there was no difference between the proportion of the red howler monkeys and the yellow-breasted capuchin monkeys that preferentially used one hand, the yellow-breasted capuchin monkeys exhibited an overall weaker hand preference than the red howler monkeys. Differences in hand preference diminished with the increasing complexity of the reaching-for-food tasks, i.e., the relatively more complex tasks were perceived as equally complex by both the red howler monkeys and the yellow-breasted capuchin monkeys. These findings suggest that species-specific differences in feeding ecology and niche structure can influence the perception of the complexity of the task and, consequently, hand preference.

Studies on hand preference in non-human primates used terms such as ‘task complexity’ and ‘task demands’ without ever comprehensively defining them–they used to measure complexity of a task typically in terms of the number of steps preceding the terminal act of reaching for food, with no reference to the precision of movement in any of the manual actions. Then, the proposition of the task complexity theory was accompanied by a working definition of complexity that took into account the precision of movements relative to the spatiotemporal dimensions of the task [1]. However, many of the preceding steps, even when requiring less precision, might not be a part of the behavioral repertoire of an individual, a population, or a species; in this case, inter-individual, -population, or -species comparisons of hand preferences across complex tasks are likely to be erroneous. In the present study, the red howler monkeys selectively took part in the reaching-for-food tasks and also exhibited stronger hand preferences than the yellow-breasted capuchin monkeys. Both these observations demonstrate the contingent nature of the complexity of a task.

Any reaching-for-food action in tripedal/quadrupedal posture is inherently bimanual, and not unimanual, since one hand is used to support the body. This inherently bimanual activity involves two kinds of dexterity: the one associated with maneuvering in three-dimensional space and the other associated with providing physical support. Hence, studies that compared the hand-usage patterns of individuals in the same unimanual or bimanual tasks that require tripedal/quadrupedal posture in one setup and bipedal posture in the other (captive red-capped mangabeys [8] and captive tufted capuchin monkeys [38]) described division of labor in hand usage as has been demonstrated in bonnet macaques (*Bonnet macaque*) [39]. Findings on captive red-capped mangabeys indicating a stronger hand preference while hanging than when sitting or quadrupedal [8] may also have reported a similar division of labor in hand usage. Findings on orangutans and chimpanzees indicating a right-hand preference only while acquiring bipedal instead of quadrupedal posture [25] overlooked these constraints and may, therefore, have presented artifacts. Other studies reported no influence of body posture on hand preference (gray mouse lemurs, *Microcebus murinus* [40], [41] and galagos, *Galago moholi* [41]), and we also obtained similar results in the present study. These findings qualify the assumptions underlying the postural origins theory, and suggest that ecological constraints could have also played a role in the evolution of hand preference in non-human primates.

An ideal setup to examine the influence of the complexity of a task on hand preference (independent of the influence of body posture) should involve unimanual or bimanual tasks that vary in complexity, but require the same body posture. Similarly, in order to examine the influence of body posture on hand preference (independent of the influence of the complexity of a task), one should employ tasks that require different body postures, but involve the same level of complexity. Unfortunately our setup did not fulfill these criteria due to insufficient data on the red howler monkeys that almost never reached for food presented on the opposite side of a wire mesh or inside a portable container, and almost never acquired bipedal and clinging posture. Besides, many of our study subjects were young, and this could have also influenced the observed hand-usage patterns as has been shown in tufted capuchin monkeys [42], but we were not able to test for such an effect due to the low sample size. However, there were no apparent differences in the hand-usage patterns between infants, juveniles, and adults. Hence, our observations should be treated with caution. Regardless of these weaknesses in the design and execution of our experiments, our findings suggest that the feeding ecology and niche structure of a species, especially with regard to the complexity of a task, are likely to have shaped the evolution of hand preference in non-human primates, though in a much more complex way. However, further studies are required to determine the importance of specific elements such as terrestriality, object manipulation, and tool use.

Although several studies have investigated the evolutionary origin of hand-preference in non-human primates, methodological differences and confounding variables were not appropriately addressed. We suggest that, to obtain more unambiguous answers, more comparative studies should be conducted, with experimental designs that allow comparing hand-usage patterns across species that vary in their phylogenetic relatedness and/or ecology, over a range of spontaneous activities and experimental tasks. It might be useful to study manual preferences not just in isolation, but within the broader scope of the behavioral repertoire of the species. Also, it might be advantageous to study the ontogeny of manual preferences. Studies of these kinds may help to understand the potential selection pressures under which hand preference has evolved.
